# Clinical Outcomes of Robotic-Assisted Gynecologic Surgery: A Single-Center 70-Case Series Experience

**DOI:** 10.7759/cureus.105309

**Published:** 2026-03-16

**Authors:** Priya Bhave, Sonal Jain, Kamal Uddin Zaidi, Rahul Gour, Madhuri Brahmne, Mrinal Chatterjee, Neetu Mehta, Narendra Thakur

**Affiliations:** 1 Reproductive Medicine, Harmony Institute of Excellence in Reproductive Health (HER Health), Bhopal, IND; 2 Gynecology, Harmony Institute of Excellence in Reproductive Health (HER Health), Bhopal, IND; 3 Biotechnology Pharmacology Laboratory and Human Genetics Laboratory, Harmony Institute of Excellence in Reproductive Health (HER Health), Bhopal, IND; 4 Centre for Scientific Research and Development, People’s University, Bhopal, IND; 5 Research, Harmony Institute of Excellence in Reproductive Health (HER Health), Bhopal, IND

**Keywords:** cystectomy, fertility preservation, gynecology, hysterectomy, minimally invasive surgery, myomectomy, robotic-assisted surgery, ssi mantra™

## Abstract

Background

Robotic-assisted surgery has emerged as an advanced minimally invasive approach in gynecology, offering enhanced visualization, precision, and ergonomics. However, real-world clinical data from Indian centers, particularly using indigenously developed robotic platforms, remain limited.

Objective

This study aims to evaluate the spectrum of indications, perioperative outcomes, and robotic-assisted gynecologic procedures performed with the SSI Mantra 3 Surgical Robotic System (SS Innovations International Inc., Gurugram, India) at a tertiary care center in India.

Methods

This retrospective case series included 70 consecutive patients who underwent robotic-assisted gynecologic surgery between August and December 2025. Procedures were performed using the SSI Mantra™ robotic system and included robotic hysterectomy (RH), robotic myomectomy (RM), and robotic cystectomy (RC) for benign gynecologic indications. Demographic variables, operative parameters (operative time, docking time, console time), estimated blood loss, length of hospital stay, and perioperative complications were analyzed using descriptive statistics.

Results

Of the 70 procedures, RH was the most common (31 cases, 44%), followed by RC (20 cases, 29%) and RM (19 cases, 27%). Patients undergoing RH were older than those in RM and RC, while body mass index was comparable across groups. RC demonstrated the shortest operative time (60.3 ± 18.5 minutes), lowest estimated blood loss (28.3 ± 39.6 mL), and shortest hospital stay (2.3 ± 0.8 days). RH had the longest operative time (119.3 ± 44.0 minutes) but remained associated with acceptable blood loss (113.8 ± 66.9 mL) and short hospitalization (2.7 ± 0.8 days). No intraoperative or postoperative complications were observed in any group.

Conclusion

Robotic-assisted gynecologic surgery using the SSI Mantra™ system is safe, feasible, and effective across a broad range of benign gynecologic conditions. The favorable perioperative outcomes, minimal blood loss, short hospital stay, and absence of complications support the integration of this indigenous robotic platform into contemporary minimally invasive gynecologic practice in India.

## Introduction

Robotic surgery is a significant advancement in minimally invasive surgery, integrating the benefits of laparoscopy with the added advantages of precision, vision, and ergonomics [1]. Robotic-assisted surgery has been gradually introduced into gynecology as a sophisticated form of minimally invasive surgery, enabling complex procedures to be performed with greater precision and control than conventional surgery [2]. Conventional open gynecologic surgery is known to be accompanied by considerable postoperative morbidity, including increased blood loss, pain, hospital stay, and functional recovery time [3]. Although laparoscopy has greatly reduced the invasiveness of surgery, its use has been limited by the rigidity of the instruments, lack of freedom of movement, two-dimensional vision, and surgeon fatigue, especially during long and complex procedures [4,5].

The robotic surgical systems were developed to overcome these challenges by providing three-dimensional high-definition visualization, multi-articulated instruments with multiple degrees of freedom, tremor elimination, and excellent ergonomic support for the surgeon [6,7]. These characteristics are very useful in gynecologic surgery, where delicate dissection, accurate suturing, and preservation of pelvic anatomy are required. Thus, robotic-assisted surgery has been widely accepted for the treatment of both benign and reproductive gynecologic disorders [8].

Hysterectomy, myomectomy, and cystectomy are the most common gynecologic procedures performed and are often complicated by complex pelvic anatomy. Robotic-assisted hysterectomy provides superior visualization of vascular pedicles and surrounding anatomy, making safe uterine excision feasible with decreased postoperative morbidity [9]. Robotic myomectomy (RM) provides precise control over fibroid resection and multilayer uterine reconstruction, which is essential for maintaining uterine integrity and fertility [10]. Similarly, robotic cystectomy (RC) provides meticulous resection of adnexal pathology with minimal trauma to ovarian tissue and surrounding anatomy, particularly in endometriosis-related disorders [11]. Prior studies have shown that robotic gynecologic surgery is characterized by minimal blood loss, low complication rates, and excellent postoperative recovery with outcomes equivalent to or surpassing those of conventional laparoscopic surgery [12,13].

Abnormal uterine bleeding in perimenopausal and postmenopausal women is a common clinical issue that may indicate underlying uterine pathology. Transvaginal B-mode ultrasound is commonly used for initial evaluation of the uterus and endometrium, but it may have limitations in differentiating intracavitary lesions. Doppler ultrasound can provide additional information by assessing blood flow patterns, potentially improving diagnostic accuracy. Therefore, Nguyen and Nguyen (2023) investigated the added value of Doppler ultrasound alongside B-mode ultrasound in detecting uterine intracavitary pathologies in women with abnormal bleeding [14].

However, the available data from real-world settings remain limited, underscoring the importance of observational studies to evaluate complication rates, conversion to open surgery, learning curves, and recovery outcomes. Patient-related outcomes, such as hospital stay and early recovery of function, are also often underreported, despite their importance.

In our study, we employed the SSI Mantra 3 Surgical Robotic System (SS Innovations International Inc., Gurugram, India), which is the first indigenous robotic system in India. It provides a more accessible and cost-effective alternative with an open console, 3D visualization, modular robotic arms, and wristed instruments. We describe our initial experience with its clinical feasibility in the management of benign and reproductive gynecologic conditions.

## Materials and methods

Study design and setting

This retrospective case series study was conducted between August and December 2025 at a tertiary care center, Harmony Institute of Excellence in Reproductive Health (HER Health Hospital). Approval for the study was obtained from the Institutional Ethics Committee (IEC No. SAIMS/IEC/12/26) prior to data collection. Written informed consent was obtained from all patients for robotic surgery and for the use of their anonymized clinical data for research purposes.

A total of 70 patients who underwent robotic gynecologic surgery during the study period were included. The study aimed to evaluate the clinical outcomes of robotic surgeries performed for benign gynecological conditions.

Data were collected retrospectively from hospital medical records, operative notes, and perioperative documentation. Adult female patients aged 18 years or older who underwent robotic hysterectomy (RH), RM, or RC were included in the study.

Patients were included only if complete clinical records were available and documented informed consent was present. Patients were excluded if they declined consent, required emergency surgery, had contraindications to minimally invasive or robotic surgery, or had incomplete clinical records.

Port positioning and docking of the robotic system (SSI Mantra™ platform)

**Figure 1:**
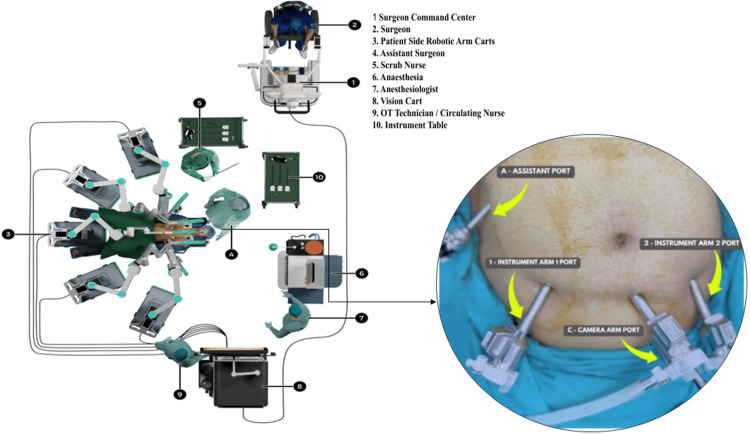
Operating room configuration and abdominal port placement for robotic gynecological surgery using the SSI Mantra robotic system. Original image by the authors.

All robotic gynecologic procedures in the current study were conducted using the SSI Mantra™ robotic surgical system according to a standardized operative technique. Under general anesthesia, patients were placed in the lithotomy position with Trendelenburg tilt for optimal pelvic access. Pneumoperitoneum was achieved with carbon dioxide insufflation, and trocar sites were arranged for the three-dimensional camera and robotic instruments (Figure 1). Following the trocar site arrangement, the robotic system was docked, and the operating surgeon proceeded to conduct the procedure from the console station with real-time three-dimensional imaging and wristed instrument dexterity.

In RH, the uterus was dissected from surrounding pelvic structures with meticulous vascular pedicle identification and control. Adnexal structures were either preserved or excised based on clinical indication. The specimen was removed, and the vaginal vault was closed with intracorporeal robotic suturing. In RM, uterine fibroids were identified and enucleated through strategically placed uterine incisions, followed by meticulous multilayer myometrial suturing to restore uterine anatomy and preserve fertility. RC entailed the excision of ovarian or pelvic cysts with meticulous preservation of surrounding structures. Upon completion of the procedure, the system was undocked, trocars were removed, and skin incisions were closed.

## Results

**Figure 2:**
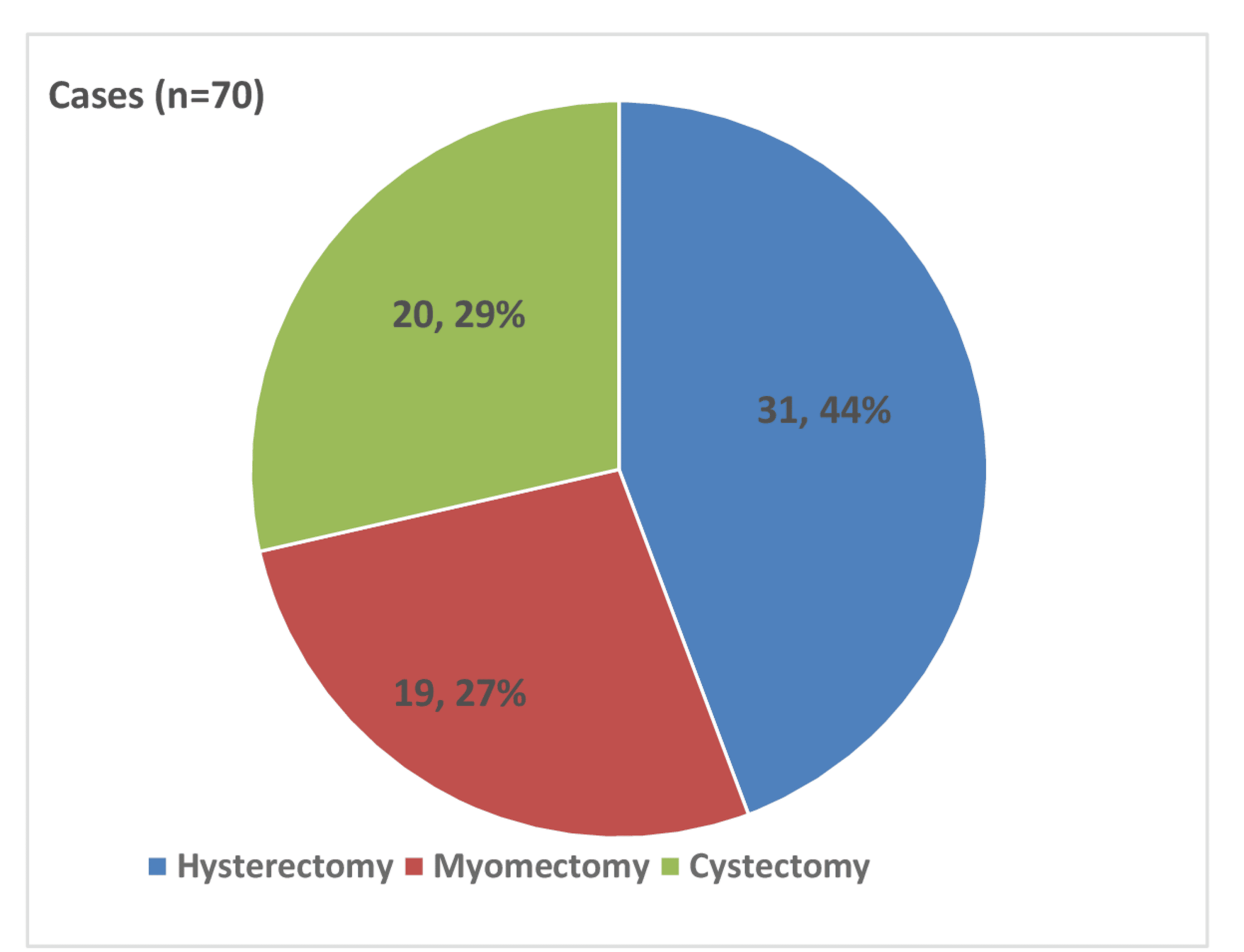
Pie chart depicting the distribution of robotic gynecological procedures performed during the study period. Robotic hysterectomy (RH) constituted the largest proportion (31, 44%), followed by myomectomy (19, 27%) and cystectomy (20, 29%).

**Figure 3:**
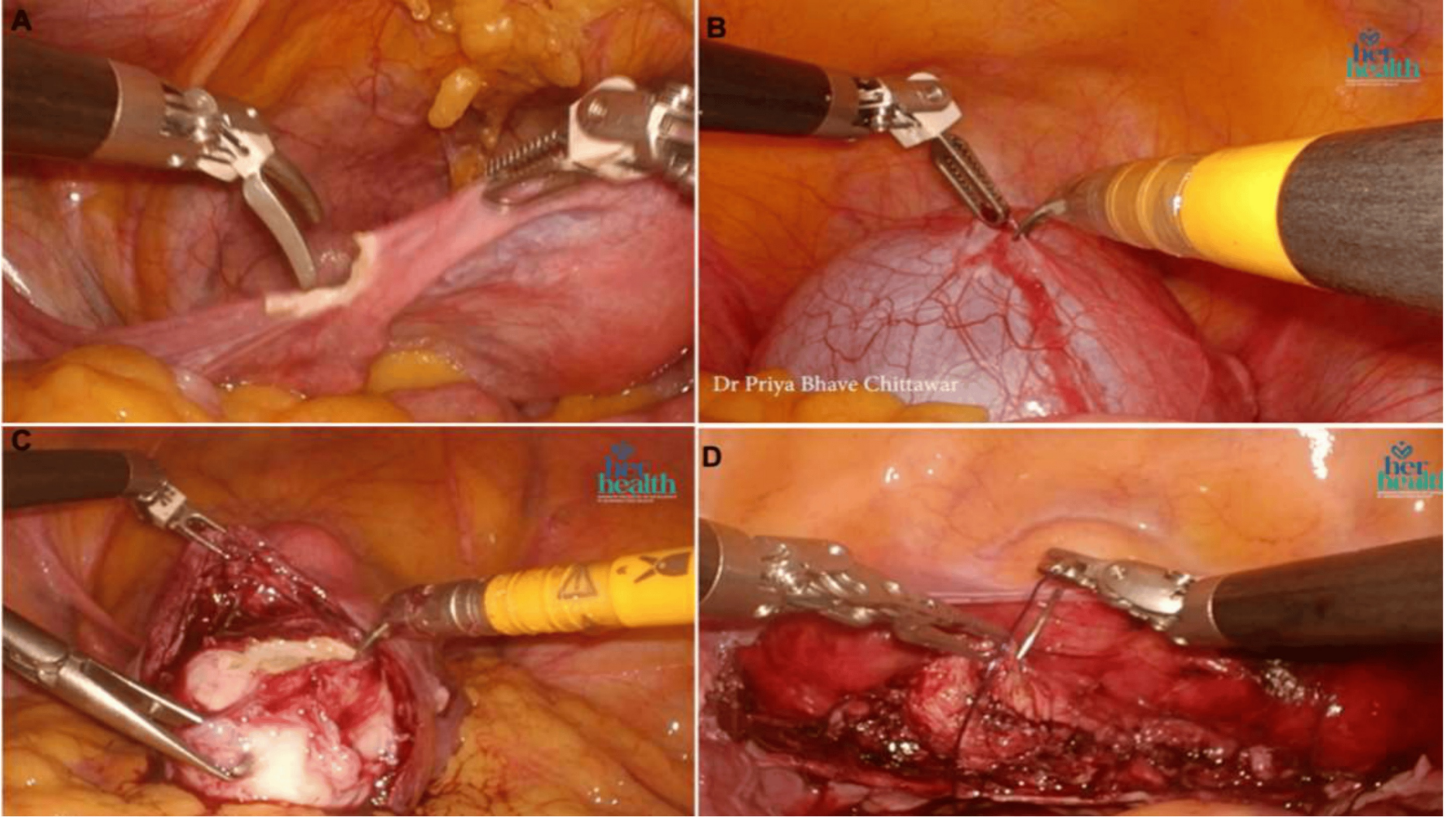
Intraoperative robotic-assisted gynecological procedures. Robotic-assisted hysterectomy demonstrated division of the cornual structures. (A) Robotic-assisted paraovarian cystectomy demonstrated precise cyst wall dissection with preservation of normal adnexal parenchyma. (B) Robotic-assisted myomectomy showed fibroid enucleation. (C) Robotic-assisted hysterectomy demonstrated vaginal vault suturing after uterine specimen removal. (D) Intraoperative image showing robotic-assisted hysterectomy demonstrating suturing of the vaginal vault after the removal of the specimen of the uterus.

A total of 70 robotic-assisted gynecologic procedures were evaluated and divided into three groups: RH, RM, and RC. RH had the highest number of procedures (31, 44%), which were primarily performed for abnormal uterine bleeding, adenomyosis, uterine prolapse, and other benign conditions of the uterus. RM was the second most common procedure (19, 27%), which was performed primarily for uterine fibroids in women requiring uterine preservation, including those with infertility. RC was performed in 20 (29%) cases, which were predominantly for endometriotic and dermoid ovarian cysts. Intraoperative images show the representative steps of each procedure (Figures 2, 3).

The study consisted of patients divided into three groups: RC (n = 19), RM (n = 18), and RH (n = 33). The mean age of the patients was 28.2 ± 3.6 years in the RC group, 34.6 ± 6.5 years in the RM group, and 44.0 ± 5.6 years in the RH group. The mean body mass index (BMI) was 24.0 ± 2.8 kg/m² for RC, 26.2 ± 3.7 kg/m² for RM, and 26.2 ± 2.5 kg/m² for RH. The operative and perioperative results for RC, RM, and RH are presented in Table 1. The mean operative time increased with each procedure, with the shortest time in RC (60.3 ± 18.5 minutes), followed by RM (83.6 ± 34.2 minutes), and finally RH (119.3 ± 44.0 minutes). The docking time was similar for all three procedures, averaging 7-8 minutes. The mean console time was similar to the total operative time for all three procedures. Vault suturing was performed only in the RH group, with a mean time of 15.7 ± 2.0 minutes. The mean postoperative hospital stay was similar for all three procedures, ranging from 2.3 to 2.7 days. The mean estimated blood loss was lowest in RC and highest in RH. The mean uterine weight in the RH group was 106.2 ± 17.6 g (Table 1).

**Table 1:** Demographic and operative and postoperative outcomes according to type of surgery. Data are presented as mean ± standard deviation for descriptive purposes only; no intergroup statistical comparison was performed. BMI (kg/m²) – Body Mass Index (kg/m^2^); RC – Robotic Cystectomy; RM – Robotic Myomectomy; RH – Robotic Hysterectomy

Variable	RC	RM	RH
Age (years)	28.2 ± 3.6	34.6 ± 6.5	44.0 ± 5.6
BMI (kg/m²)	24.0 ± 2.8	26.2 ± 3.7	26.2 ± 2.5
Total operative time (min)	60.3 ± 18.5	83.6 ± 34.2	119.3 ± 44.0
Docking time (min)	7.2 ± 2.0	7.6 ± 1.3	7.8 ± 2.8
Console time (min)	60.3 ± 18.5	83.6 ± 34.2	119.3 ± 44.0
Vault suture time (min)	NA	NA	15.7 ± 2.0
Post-op hospital stay (days)	2.3 ± 0.8	2.6 ± 0.6	2.7 ± 0.8
Estimated blood loss (mL)	28.3 ± 39.6	78.6 ± 115.2	113.8 ± 66.9
Uterus weight (g)	NA	NA	106.2 ± 17.6

## Discussion

Robotic-assisted surgery has become a standard method in minimally invasive gynecology, especially for treating complicated benign and reproductive issues. Robotic platforms provide superior three-dimensional visualization, enhanced instrument articulation, tremor filtration, and improved surgeon ergonomics compared to conventional laparoscopy. These features facilitate precise dissection and suturing in challenging scenarios, including enlarged uteri, deep pelvic disease, and dense adhesions [15,16]. Gynecological disorders, such as uterine fibroids, endometriosis, adenomyosis, and cervical premalignant lesions, are frequently observed and carry considerable reproductive, physical, and quality-of-life consequences. Uterine fibroids (leiomyomas) are the most common benign tumors in women of reproductive age. Surgical management is tailored based on symptom severity, disease characteristics, and patient preferences, with myomectomy or hysterectomy considered suitable options when conservative treatment fails [17,18].

In this study, patients undergoing robotic-assisted surgery for benign gynecological conditions have their key perioperative parameters evaluated. The demographic profile is consistent with that of the general population receiving minimally invasive treatment for benign pelvic pathology, as indicated by the mean age and BMI. Previous robotic gynecological series have reported similar age distributions and BMI ranges, suggesting that robotic platforms can be used with a wide range of reproductive and perimenopausal women without significant demographic restrictions [19,20]. When vaginal or laparoscopic methods are not feasible, the use of a robotic platform has been demonstrated to improve postoperative outcomes for patients undergoing benign hysterectomy, particularly for those with a high BMI [21].

Operative efficiency in our study, assessed by total operative time, demonstrates acceptable procedural duration with stable robotic integration. The mean total operative time was 60.3 ± 18.5 minutes in the RC group, 83.6 ± 34.2 minutes in the RM group, and 119.3 ± 44.0 minutes in the RH group. The total operative time observed in our cohort falls within the range reported in the literature, where operative duration varies depending on surgical complexity and surgeon experience [22]. A previous study that compared the outcome of RH with LH for large uteri in Indian women also showed a longer operative time (131.0 minutes) [23]. Furthermore, another study conducted in India on benign pathologies also revealed a longer mean total operative time of 127.37 ± 110.67 minutes for RM [24]. The longer operating times in robotic procedures may be due to the time required to set up, position, dock, and undock the robotic system before surgery, as reported in various studies.

Docking time remained relatively short in our cohort, with a mean docking time of 7.2 ± 2.0 minutes in the RC group, 7.6 ± 1.3 minutes in the RM group, and 7.8 ± 2.8 minutes in the RH group, indicating procedural familiarity and streamlined operating room workflow, findings that are consistent with previously documented learning-curve improvements in robotic surgery [25]. Furthermore, research has demonstrated that robot docking durations are longer in the early learning phase and much shorter as operating experience increases [26]. Console time constituted a significant component of operative duration, which aligns with the nature of robotic surgery, where intracorporeal dissection and suturing are performed under enhanced three-dimensional visualization. In a single-center study in India, real-time data from a decade of robotic surgery for benign gynecological disorders were analyzed. The study concluded that docking time, console time, and operating time improved significantly and consistently over time.

Estimated blood loss in this study was low overall, with a mean blood loss of 28.3 ± 39.6 mL in the RC group, 78.6 ± 115.2 mL in the RM group, and 113.8 ± 66.9 mL in the RH group, supporting the hemostatic advantage often attributed to robotic instrumentation. Improved magnification, tremor filtration, and articulated wrist instruments allow precise tissue handling and vascular control, which may reduce intraoperative bleeding. Comparable blood loss outcomes have been described in earlier reports of robotic surgery for benign gynecological conditions [27]. Postoperative hospital stay was short in our cohort, with a mean duration of 2.3 ± 0.8 days in the RC group, 2.6 ± 0.6 days in the RM group, and 2.7 ± 0.8 days in the RH group, reflecting early postoperative recovery and minimal perioperative morbidity. These findings are consistent with established minimally invasive surgical principles, in which reduced tissue trauma and smaller incisions facilitate faster mobilization and discharge [28].

In RH, the uterus weight, where applicable, represented the benign disease spectrum managed in this series, with a mean uterine weight of 106.2 ± 17.6 grams. Previous studies have demonstrated that robotic technology allows safe surgical management across varying uterine sizes without significantly increasing complication rates [29]. Overall, the perioperative parameters observed in this study support the safety, feasibility, and reproducibility of robotic-assisted surgery in benign gynecology. Consistent operative metrics, low blood loss, and short hospitalization reinforce the role of robotic technology as an effective minimally invasive surgical modality.

## Conclusions

Robotic-assisted gynecologic surgery using the SSI Mantra™ system is a safe and effective treatment option for benign gynecologic diseases. The procedures were associated with minimal blood loss, short hospital stays, and favorable perioperative outcomes. These results support the adoption of robotic-assisted surgery in modern minimally invasive gynecologic surgery in India.
